# Exercise‐induced peptide EIP‐22 protect myocardial from ischaemia/reperfusion injury via activating JAK2/STAT3 signalling pathway

**DOI:** 10.1111/jcmm.16441

**Published:** 2021-03-12

**Authors:** Li Zhang, Xuejun Wang, Hao Zhang, Mengwen Feng, Jingjing Ding, Bing Zhang, Zijie Cheng, Lingmei Qian

**Affiliations:** ^1^ Department of Cardiology Tongren Hospital Shanghai Jiao Tong University School of Medicine Shanghai China; ^2^ Department of Cardiology The First Affiliated Hospital of Nanjing Medical University Nanjing China; ^3^ Department of Internal Medicine The Affiliated Drum Tower Hospital of Nanjing University Medical School Nanjing China; ^4^ Department of General Practice Tongren Hospital Shanghai Jiao Tong University School of Medicine Shanghai China; ^5^ Hongqiao International Institute of Medicine Tongren Hospital Shanghai Jiao Tong University School of Medicine Shanghai China

**Keywords:** EIP‐22, JAK2/STAT3, myocardial I/R, peptide

## Abstract

Recent studies have revealed that exercise has myocardial protective effects, but the exact mechanism remains unclear. Studies have increasingly found that peptides play a protective role in myocardial ischaemia‐reperfusion (I/R) injury. However, little is known about the role of exercise‐induced peptides in myocardial I/R injury. To elucidate the effect of exercise‐induced peptide EIP‐22 in myocardial I/R injury, we first determined the effect of EIP‐22 on hypoxia/reperfusion (H/R)‐ or H_2_O_2_‐induced injury via assessing cell viability and lactate dehydrogenase (LDH) level. In addition, reactive oxygen species (ROS) accumulation and mitochondrial membrane potential (MMP) was assessed by fluorescence microscope. Meanwhile, Western blot and TUNEL methods were used to detect apoptosis level. Then, we conducted mice I/R injury model and verified the effect of EIP‐22 by measuring cardiac function, evaluating heart pathology and detecting serum LDH, CK‐MB and cTnI level. Finally, the main signalling pathway was analysed by RNA‐seq. In vitro, EIP‐22 treatment significantly improved cells viabilities and MMP and attenuated the LDH, ROS and apoptosis level. In vivo, EIP‐22 distinctly improved cardiac function, ameliorated myocardial infarction area and fibrosis and decreased serum LDH, CK‐MB and cTnI level. Mechanistically, JAK/STAT signalling pathway was focussed by RNA‐seq and we confirmed that EIP‐22 up‐regulated the expression of p‐JAK2 and p‐STAT3. Moreover, AG490, a selective inhibitor of JAK2/STAT3, eliminated the protective roles of EIP‐22. The results uncovered that exercise‐induced peptide EIP‐22 protected cardiomyocytes from myocardial I/R injury via activating JAK2/STAT3 signalling pathway and might be a new candidate molecule for the treatment of myocardial I/R injury.

## 1 INTRODUCTION

Ischaemic heart disease is a type of cardiovascular disease with high morbidity and high fatality rate. The annual death toll is nearly 17.8 million, which has long occupied the top of various lethal factors.^1^ Although interventional surgery has relieved clinical symptoms to a certain extent and improved the survival rate of patients with ischaemic heart disease, the myocardial injury caused by ischaemia‐reperfusion (I/R) is a difficult and cutting‐edge problem in the field of cardiovascular disease prevention and treatment,^2^ which needs to be solved urgently. Numerous studies have shown that cardiomyocytes apoptosis and reactive oxygen species (ROS) accumulation are strongly associated with myocardial ischaemia‐reperfusion injury.^3^, ^4^, ^5^ However, there is no effective method to protect myocardial from ischaemia‐reperfusion induced injury.

Accumulating evidence suggested that moderate exercise can prevent and improve cardiovascular disease.^6^ Exercise training can reduce long‐term complications and improve the quality of survival after myocardial infarction.^7^, ^8^ Moderate exercise can alleviate myocardial ischaemia‐reperfusion injury and has myocardial protective effects.^9^, ^10^ Recent studies have shown that some beneficial bioactive substances, such as irisin, are secreted in response to exercise stimuli, mediating the protective effects of exercise through anti‐apoptotic, antioxidant and anti‐inflammatory effects.^11^, ^12^ However, the exact mechanism of the cardioprotective effect of exercise remains unclear. Further study of the specific mechanisms by which exercise protects the heart will hopefully lead to the development of new cardioprotective drugs.

Various peptides have been shown to be involved in cardiovascular‐related pathophysiological processes and have been found to be promising drugs for the treatment of cardiovascular diseases, such as brain natriuretic peptide (BNP),^13^ Apelin‐13^14^ and glucagon‐like peptide‐1 (GLP‐1).^15^ In recent years, peptide drugs, which have the advantages of small molecular weight, low toxicity, low immune response and high biological activity, have been widely used in clinical disease treatment.^16^ These reports indicate that peptides may be effective molecules in the treatment of cardiovascular disease. In a recent study, differentially expressed peptides were identified in the peripheral plasma of four healthy male volunteers in the last minute of exercise and 1, 2 and 5 hours after exercise.^17^ A total of 425 peptides were significantly regulated during or in the immediate hours after exercise (*P* < .05; ANOVA with permutation‐based correction), of which a peptide, we named as EIP‐22 (exercise‐induced peptide, 22 amino acids), that was up‐regulated during exercise and had a high bioactivity prediction score (Peptide Ranker score is 0.80, greater than 0.5 indicate potential bioactivity) caught our attention.

Janus kinase (JAK)‐signal transducer and activator of transcription (STAT) has been confirmed to be involved in the various pathophysiological response and play a key role in the development of cardiovascular diseases.^18^, ^19^ Janus kinase 2‐signal transducer and activator of transcription 3 (JAK2/STAT3) is the most deeply studied signal pathway in JAK/STAT family. Growing evidence suggests that the JAK2/STAT3 signalling pathway plays a key role in mediating protection of myocardium from I/R injury.^20^ Therefore, targeting the JAK2/STAT3 pathway will potentially hold a certain promise for the treatment of myocardial I/R injury.

Our study aims to evaluate the role and mechanism of a novel exercise‐induced peptide EIP‐22 in myocardial I/R injury. In this study, we proved that EIP‐22 has cardioprotective effects against myocardial I/R injury in vitro and vivo through increasing cell viabilities, reducing apoptosis and ROS accumulation, improving cardiac function and ameliorating myocardial infarction area and fibrosis. In addition, we provided evidence that EIP‐22 up‐regulated the expression of phosphorylated JAK2 (p‐JAK2) and phosphorylated STAT3 (p‐STAT3) in H/R and H_2_O_2_ model, and AG490, a selective inhibitor of JAK2/STAT3, eliminated the protective roles of EIP‐22. In general, for the first time, we demonstrated the role and mechanism of a novel exercise‐induced peptide EIP‐22 in myocardial I/R injury and provided a new insight into treatment strategy of myocardial I/R injury.

## 2 MATERIALS AND METHODS

### 2.1 Drugs and reagents

The sources of antibodies utilized in this study were as listed follow: anti‐PARP (#9542), anti‐cleaved‐casepase3 (#9661), anti‐Bax (#2772), anti‐p‐JAK2 (#3776), anti‐β‐actin (#4970), anti‐GAPDH (#2118), goat anti‐rabbit and goat anti‐mouse were purchased from Cell Signaling Technology; anti‐Bcl2 (sc‐7382), anti‐JAK2 (sc‐390539), anti‐STAT3 (sc‐8019) and anti‐p‐STAT3 (sc‐8059) were purchased from Santa Cruz. The AG490, a selective inhibitor of JAK2/STAT3, was purchased from Sigma Chemical Co.

The sequence of EIP‐22 peptide was GESEAPAPPGPGTRWPYRSRDT, and the scramble peptide was WEGASPPTRAGPESGDYRPRTP (Scr). These peptides were synthesized by Shanghai Science Peptide Biological Technology Co., Ltd. The purity of these peptides was more than 95%.

The Cell Counting Kit‐8 (CCK‐8), Reactive Oxygen Species (ROS) Assay Kit, JC‐1 Mitochondrial Membrane Potential (MMP) Assay Kit were purchased from Beyotime Institute of Biotechnology; The LDH Release Assay Kit and the kits for detecting superoxide dismutase (SOD) activity, malondialdehyde (MDA) concentration, glutathione peroxidase (GSH‐Px) activity, CK‐MB and cTnI level were purchased from Jiancheng Bioengineering Institute.

### 2.2 Cell culture, hypoxic stimulation and experimental design in vitro

The cardiomyocytes were extracted from 1 to 3 days SD rats. After ventricular tissues were collected, cut and washed under aseptic conditions, 0.1% trypsin was added for digestion at 37℃ for 20 minutes, repeated 10 times. The supernatant was collected and transferred to a centrifuge tube containing 15 mL of 10% foetal bovine serum (FBS) for termination of pancreatin digestion. Next, the supernatant was centrifuged at 1000 r/min for 5 minutes at room temperature and resuspended with the appropriate DMEM medium. The collected cells were cultured in 37℃ and 5% CO_2_ incubator for 2 hours, and the myofibroblasts were removed by differential attachment. The differential attachment of myofibroblast means to remove myofibroblasts by using the different time of adhesion between cardiomyocytes and myofibroblasts. After 2 hours, myofibroblasts were almost completely adherent, but cardiomyocytes were not adherent. The cardiomyocytes in suspension were seeded on gelatin‐coated (Sigma‐Aldrich) culture plates, and then, cells were cultured on gel plates for 24‐48 hours before further experiments. The cardiomyocytes were cultured in DMEM medium (Gibco) supplemented with 10% FBS (Gibco) and 1% penicillin/streptomycin (Wisent, Canada) in an incubator with 95% air and 5% CO_2_ at 37℃.

The cardiomyocytes were treated with 200 µM H_2_O_2_ for 24 hours to establish a hypoxia model. The peptides were added to the cell culture medium for 1 hour before H_2_O_2_ treatment.

For the H/R model, cardiomyocyte culture medium was converted into H/R buffer (4 mM HEPES, 12 mM KCl, 117 mM NaCl, 0.49 mM MgCl_2_, 0.9 mM CaCl_2_, 20 mM sodium lactate, 5.6 mM 2‐deoxy‐glucose, pH 6.2) and placed in a hypoxia chamber (95% N2/5% CO_2_, Billups‐Rothenberg) at 37℃ for 30 minutes. The chamber was closed, and normal oxygen supply was given for an additional 4 hours, followed by reperfusion with DMEM medium supplemented with 10% FBS.^21^ The peptides were added to the cell culture medium during reperfusion.

### 2.3 Detection of cell viability

CCK‐8 kit was used to detect cell viability according to the following steps. The cardiomyocytes (5 × 10^3^ /well) were seeded in 96‐well plates, and upon reaching the adherence stage, the cells were treated with drugs as described above. 10 µL CCK‐8 reagent was added to each well and cultured away from light in 37℃ incubators. After 2 hours, a microplate reader was used to measure the intensity of the light absorption at 450 nm wavelength. In brief, we first calculated the number of inoculated cells in each group to ensure that the number of cells in each group is basically equal and then measured the OD_450nm_ value of each group after drugs treatment. The OD_450nm_ value was normalized by the number of inoculated cells. Cell viability was calculated with the normalized OD ratio of experimental and control well (Cell viability = OD_450nm_ experiment / OD_450nm_ control).

### 2.4 Detection of LDH

Levels of LDH released were detected in the cell supernatant or mice serum using an LDH release assay kit (Nanjing Jian) according to the manufacturer's instruction. The cardiomyocytes (5 × 10^3^ /well) were calculated and seeded in 96‐well plates and were treated with drugs as described above. The cell supernatant or serum (120 µL/well) were collected and mixed with reaction solution (60 µL/well), and then, the mixtures were added into 96 well plates. The plate was maintained away from light for 30 minutes at room temperature on the shaker. Finally, a microplate reader was used to measure the intensity of the absorbance at OD490nm wavelength. The OD_490nm_ value was normalized by the number of inoculated cells in each group.

### 2.5 Detection of ROS, MDA, SOD and GSH‐Px

The cardiomyocytes were plated in 6‐well plates at a density of 5 × 10^5^ per well and treated as described above. The levels of intracellular ROS were determined using a ROS assay kit following the manufacturer's instruction. The cells were incubated in serum‐free DMEM medium including 0.1% DCFH‐DA at 37°C for 20 minutes and then washed with serum‐free DMEM three times. A fluorescence microscope (BX61; Olympus Corporation) was used to photograph. The ROS fluorescence was quantitated by Image J software 1.26. MDA, SOD and GSH‐Px assay kits were used to detect intracellular MDA, SOD and GSH‐Px levels according to the manufacturer's protocol.

### 2.6 Determination of mitochondrial membrane potential

The mitochondrial membrane potential was measured by a JC‐1 assay kit according to the manufacturer's instructions. The cardiomyocytes were plated in 6‐well plates at a density of 5 × 10^5^ per well and treated as described above. After treatment, the cardiomyocytes were cultured with serum‐free DMEM medium including (1×) JC‐1 staining working fluid at 37°C for 20 minutes. Then, the cells were washed twice with JC‐1 buffer, and then 2 mL DMEM medium was added. The cardiomyocytes were photographed by a fluorescence microscope (BX61; Olympus Corporation) and quantitated by Image J software 1.26. JC‐1 aggregates glow red fluorescence and monomers glow green.

### 2.7 TUNEL staining

The cardiomyocytes (5 × 10^5^ /well) were seeded in 6‐well plates. After H/R treatment, the cardiomyocytes were washed twice with PBS and fixed with 4% paraformaldehyde. TUNEL staining (Promega) was used to visualize apoptotic cells following the manufacturer's instructions. The percentage of positive cells was calculated by TUNEL fluorescence density/DAPI fluorescence density, and the density was analysed using ImageJ software 1.26.

### 2.8 Western blot analysis

The protein in the cardiomyocytes was extracted with lysis buffer (including RIPA and 1% PMSF) and quantified by a BCA assay kit (23229; Thermo Fisher Scientific). 1 × SDS loading buffer was added the protein samples and denatured by boiling at 95℃ for 5 minutes. After cooling on ice, the equivalent amounts of protein samples (20 mg) were separated by 8%‐10% SDS‐PAGE gel and then transferred to PVDF membranes (Millipore). The membranes were blocked with 5% skimmed milk at room temperature and then incubated with the corresponding specific antibody overnight at 4°C. The membranes were washed three times with TBST buffer. After that, the membranes were incubated with horseradish peroxidase‐combined secondary antibodies (anti‐rabbit/mouse) for 1 hour at room temperature. Protein expression was quantitatively analysed by Image Lab software (Bio‐Rad). Western blot results were normalized by β‐actin.

### 2.9 Mice and I/R model

The male C57BL/6 mice, 8 week‐old, were purchased from Shanghai Slake Experimental Animal Co., Ltd., and raised at the SPF Laboratory Animal Center of Nanjing Medical University. The mice were fed adaptively for one week. All mice experiments were conducted according to the Guide for the Care and Use of Laboratory Animals published by the National Institutes of Health (NIH Publications No. 85‐23, revised 1996) and reviewed by the Animal Experiment Ethics Committee of Nanjing Medical University (Nanjing, China).

The model of I/R injury was established by 45 minutes ischaemia and 1 week reperfusion.^22^ Before I/R injury surgery, mice were anaesthetized by intraperitoneal injection of chloral hydrate. Then, the chest was opened and the heart was exposed to determine the position of the left anterior descending (LAD) coronary artery. The LAD was ligated with 6‐0 silk thread for myocardial ischaemia. After 45 minutes of ischaemia, the ligation was released and the heart was reperfused for 1 week. The whole procedure was used in the sham operation without LAD ligation and release. The experimental mice were randomly allocated to four groups: the sham with Scr peptide group, the sham with EIP‐22 peptide group, the I/R with Scr peptide group and the I/R with EIP‐22 peptide group, and 12 mice in each group. The peptide EIP‐22 or Scr were dissolved in normal saline and injected into the tail vein before reperfusion. The dosage of peptide (EIP‐22 or Scr) was 10 mg/kg.

### 2.10 Echocardiography

After a week reperfusion, all mice were anaesthetized lightly with 1.5% isoflurane and allowed to breathe spontaneously, and then, mice cardiac function was detected using high‐resolution small animal ultrasound imaging system (Vevo 3100). The main measurement indicators included ejection fraction (EF), fractional shortening (FS), left ventricular end‐systolic diameter (LVEDs) and left ventricular end‐diastolic diameter (LVEDd). The fractional shortening (FS%) was calculated as [(LVEDd‐LVEDs) / LVEDd] × 100; ejection fraction (EF%) was calculated as [(LVEDV‐LVESV) / LVEDV] × 100, LVEDV = [7 LVEDd^3^ / (2.4 + LVEDd)], and LVESV = [7 LVEDs^3^ / (2.4 + LVEDs)].

### 2.11 Evans blue‐TTC double staining

After a week reperfusion, the LAD coronary artery was religated, and 2 mL 2% Evans blue was injected intravenously to delineate the area at risk (AAR). Then, the heart was flushed with normal saline and frozen. After 30 minutes of cold storage at ‐20℃, it was cut into 1‐2 mm thick myocardial sections along the long axis of the heart. The sections were incubated in 1% of 2,3,5‐Triphenyltetrazolium chloride (TTC) at 37℃ for 30 minutes to delineate the myocardial infarct area (MI). The area of AAR and MI were measured using NIH Image J software.

### 2.12 Masson staining

After the ventricular tissue was harvested, immediately fixed in 4% paraformaldehyde for 48 hours, the tissue samples were de‐hydrated, paraffin embedded and sectioned into 5 µm thick slices using a sliding microtome (Leica, Nussloch). Then, the tissue sections were dewaxed, rehydrated and stained with Masson's trichrome. Lastly, the microscope was used to observe the degree of myocardial fibrosis. Masson staining was quantified using ImageJ software.

### 2.13 Detection of CK‐MB and c‐TnI

After reperfusion 2 hours, about 2 mL blood was taken from abdominal aorta and centrifuged at 4℃ for 10 minutes at 4000 r/min. After centrifugation, the serum was collected and stored in the ultra‐low temperature refrigerator for the detection of CK‐MB and cTnI level. The detection instrument was enzyme‐labelled instrument. All operations were carried out strictly according to the instructions provided by the kit (Nanjing Jiancheng Biocompany).

### 2.14 RNA sequence and KEGG pathway analysis

After cardiomyocytes were treated with EIP‐22 (*n* = 3) or scramble peptide (*n* = 3) in H/R model, the total RNA was isolated by RNA extraction kit (Qiagen, GmBH). Then, according to Illumina User Guide, the samples were analysed by RNA‐seq using an Agilent 2100 biological analyser. The data were analysed by Illumina's data acquisition software. The volcano plot and heat map were analysed by R Studio software (version 3.5.1). The KEGG pathways analysis was performed by Functional Annotation Tool DAVID Bioinformatics Resources 6.8 (https://david.ncifcrf.gov/).

### 2.15 Statistical analysis

The experimental data and statistical graphs were analysed using GraphPad Prism 8 software. The data are presented as the means ± standard deviation (SD). Statistical differences were measured with an unpaired 2‐sided Student *t* test or two‐way ANOVA with Bonferroni correction for multiple comparisons. When the *P*‐value was < .05, the difference was considered significant.

## 3 RESULTS

### 3.1 EIP‐22 increased cell viability and reduced LDH release in cardiomyocytes exposed to H/R and H_2_O_2_

**FIGURE 1:**
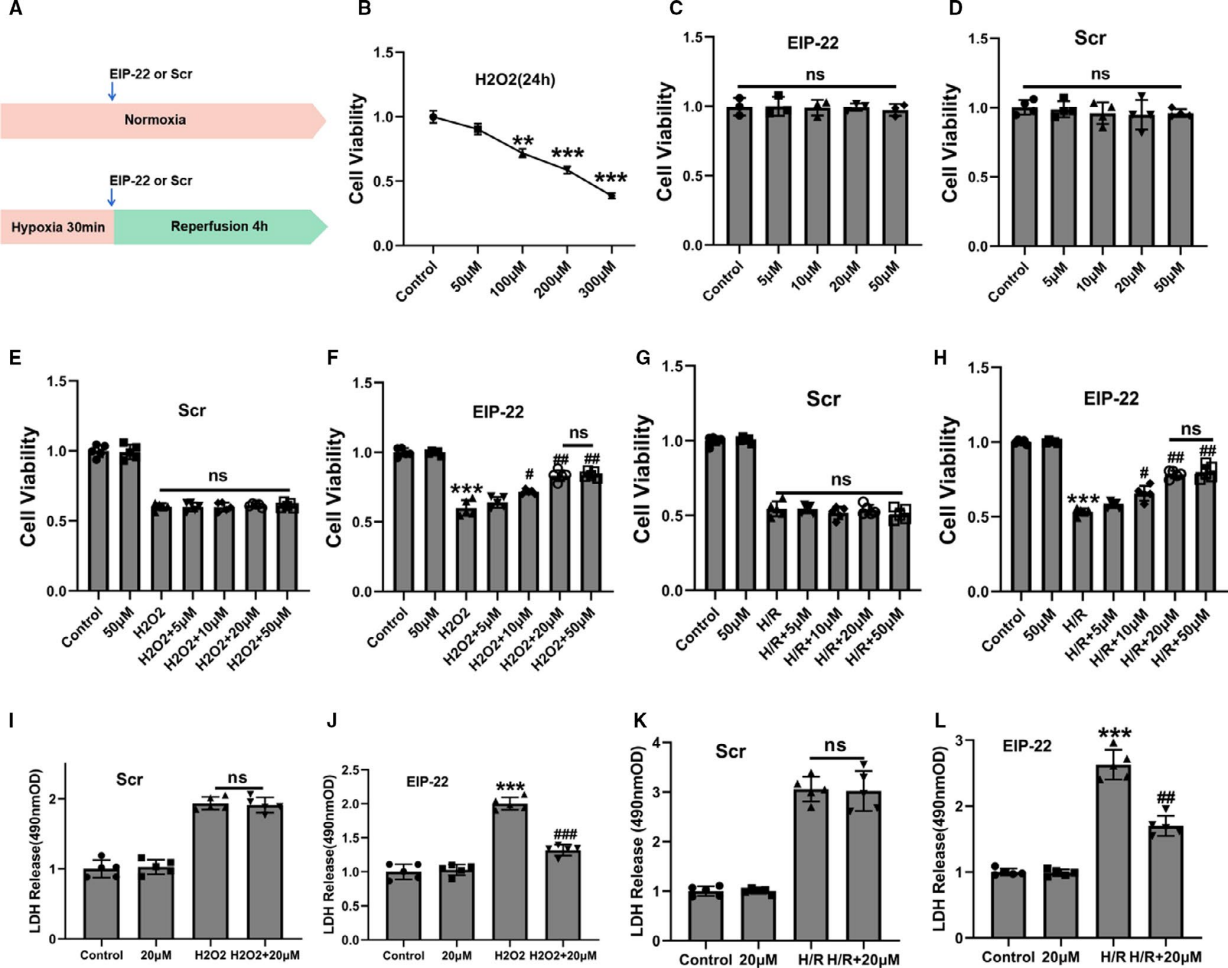
EIP‐22 improved cell viability and reduced LDH release in cardiomyocytes exposed to H/R and H_2_O_2_. A, Schematic diagram of H/R model in cardiomyocytes. B, The changes of cell viability in cardiomyocytes were treated with different concentrations of H_2_O_2_ for 24 h (50, 100, 200, 300 μM). C, Effects of different concentrations of EIP‐22 on cardiomyocytes viability (5, 10, 20, 50 μM). D, Effects of different concentrations of Scramble peptide (Scr) on cardiomyocytes viability (5, 10, 20, 50 μM). E, Effects of different concentrations of Scr on cell viability in H_2_O_2_ model. F, Effects of different concentrations of EIP‐22 on cell viability in H_2_O_2_ model. G, Effects of different concentrations of Scr on cell viability in H/R model. H, Effects of different concentrations of EIP‐22 on cell viability in H/R model. I, Effects of 20 μM Scr on LDH release in H_2_O_2_ model. J, Effects of 20 μM EIP‐22 on LDH release in H_2_O_2_ model. K, Effects of 20 μM Scr on LDH release in H/R model. L, Effects of 20 μM EIP‐22 on LDH release in H/R model. The data represent means ± SD. ^**^ *P* < .01 vs. the control group, ^***^ *P* < .001 vs. the control group, ^##^ *P* < .01 vs. the H_2_O_2_ or H/R group, ^###^ *P* < .001 vs. the H_2_O_2_ or H/R group, ns, not statistically significant

To study the function of EIP‐22, we constructed the model of H/R and H_2_O_2_ in cardiomyocytes. The schematic map of H/R model was shown in Figure 1A. Cell viability also was evaluated in cardiomyocytes treated with H_2_O_2_ at different concentrations (0, 50, 100, 200, 300 µM) for 24 hours before demonstrating the function of EIP‐22 in H_2_O_2_‐induced cardiomyocyte injury. As shown in Figure 1B, we constructed a stable H_2_O_2_ model by 200 µM H_2_O_2_ treatment for 24 hours in vitro. Subsequently, EIP‐22 peptide and its scramble peptide had no effect on cardiomyocytes without H/R and H_2_O_2_ treatment, as demonstrated by cell viability (Figure 1C,D). In addition, we assessed different concentrations of EIP‐22 and its scramble peptide can influence cell viability. As a control peptide, the scramble peptide had no effect on cell viability with H_2_O_2_ and H/R treatment (Figure 1E,G). However, EIP‐22 could increase the cell viability of cardiomyocytes in a concentration dependent manner, whether treated with H/R or H_2_O_2_, and the effect is best at 20 µM (Figure 1F,H). Next, we demonstrated that 20 µM EIP‐22 could reduce LDH release in H_2_O_2_ and H/R models (Figure 1J,L), whereas the scramble peptide had no effect (Figure 1I,K). The above results indicated that EIP‐22 exerts protection in H/R‐ and H_2_O_2_‐induced cardiomyocytes damage.

### 3.2 EIP‐22 attenuates oxidative stress and apoptosis in H/R model

**FIGURE 2:**
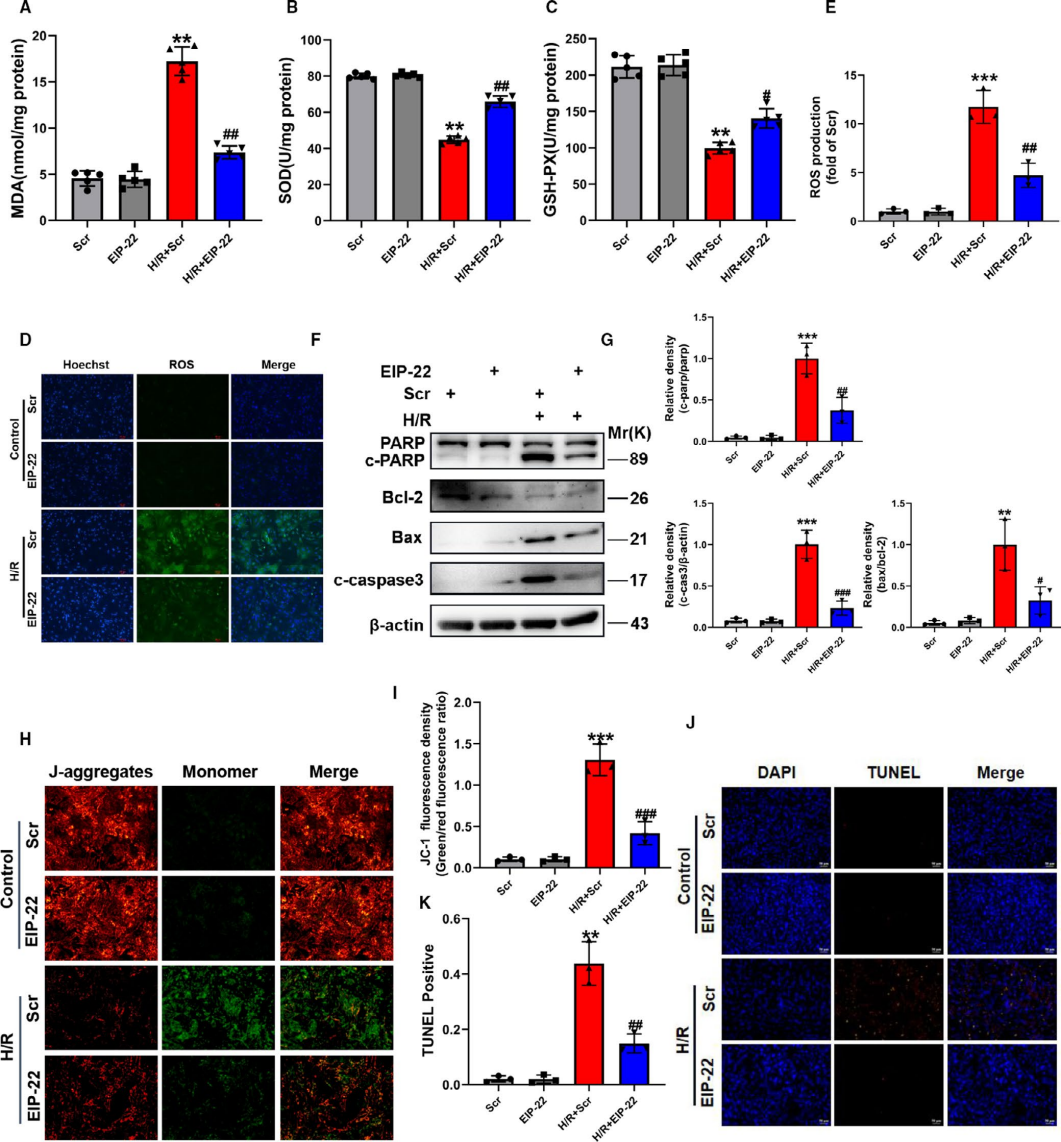
EIP‐22 attenuates oxidative stress and apoptosis in H/R model. A, Cellular MDA content in H/R model. B, Cellular SOD activities in H/R model. C, Cellular GSH‐Px activities in H/R model. D, Representative images of ROS staining in H/R model. DCFH‐DA was used to detect intracellular ROS. Green, ROS. Blue, Hoechst. E, Quantification analysis of ROS. F, The proteins related to apoptosis (PARP, cleaved caspase‐3, Bax, Bcl‐2) were measured by Western blot in H/R model. G, Quantitative analysis of relative protein expression of PARP, cleaved‐caspase, Bax and Bcl‐2. H, Representative images of mitochondrial membrane potential in H/R model. I, Quantification analysis of JC‐1 fluorescence intensity. J, Representative images of TUNEL staining in H/R model. K, Quantification analysis of TUNEL. The data represent means ± SD. ^**^ *P* < .01 vs. the Scr group, ^***^ *P* < .001 vs. the Scr group, ^#^ *P* < .05 vs. the H/R + Scr group, ^##^ *P* < .01 vs. the H/R + Scr group, ^###^ *P* < .001 vs. the H/R + Scr group

To explore the effect of EIP‐22 peptide on H/R‐induced oxidative stress in the cardiomyocytes, firstly, we measured the activities of major antioxidant enzymes, superoxide dismutase (SOD) and glutathione peroxidase (GSH‐Px), and the content of malondialdehyde (MDA) in the cardiomyocytes in H/R model. 20 µM EIP‐22 peptide significantly reduced the content of MDA (Figure 2A) and increased the activities of SOD and GSH‐Px in H/R model (Figure 2B,C). Simultaneously, ROS production in cardiomyocytes was detected by DCFH‐DA staining. The result showed that 20 µM EIP‐22 peptide significantly reduced the production of ROS in H/R model (Figure 2D,E). Apoptosis is a kind of programmed cell death, which is cascaded by many molecules, and plays a key role in myocardial injury induced by H/R. To further explore the function of EIP‐22 in H/R‐induced myocardial injury, apoptosis was evaluated. Decreased cleaved‐PARP activation, cleaved caspase‐3 and Bax level and increased Bcl‐2 level were detected after EIP‐22 peptide treatment in H/R model (Figure 2F,G). In addition, we also proved that EIP‐22 could maintain the mitochondrial membrane potential and reduce cell apoptosis rates, as indicated by JC‐1 and TUNEL assay (Figure 2H‐K).

### 3.3 EIP‐22 attenuates oxidative stress and apoptosis in H_2_O_2_ model

**FIGURE 3:**
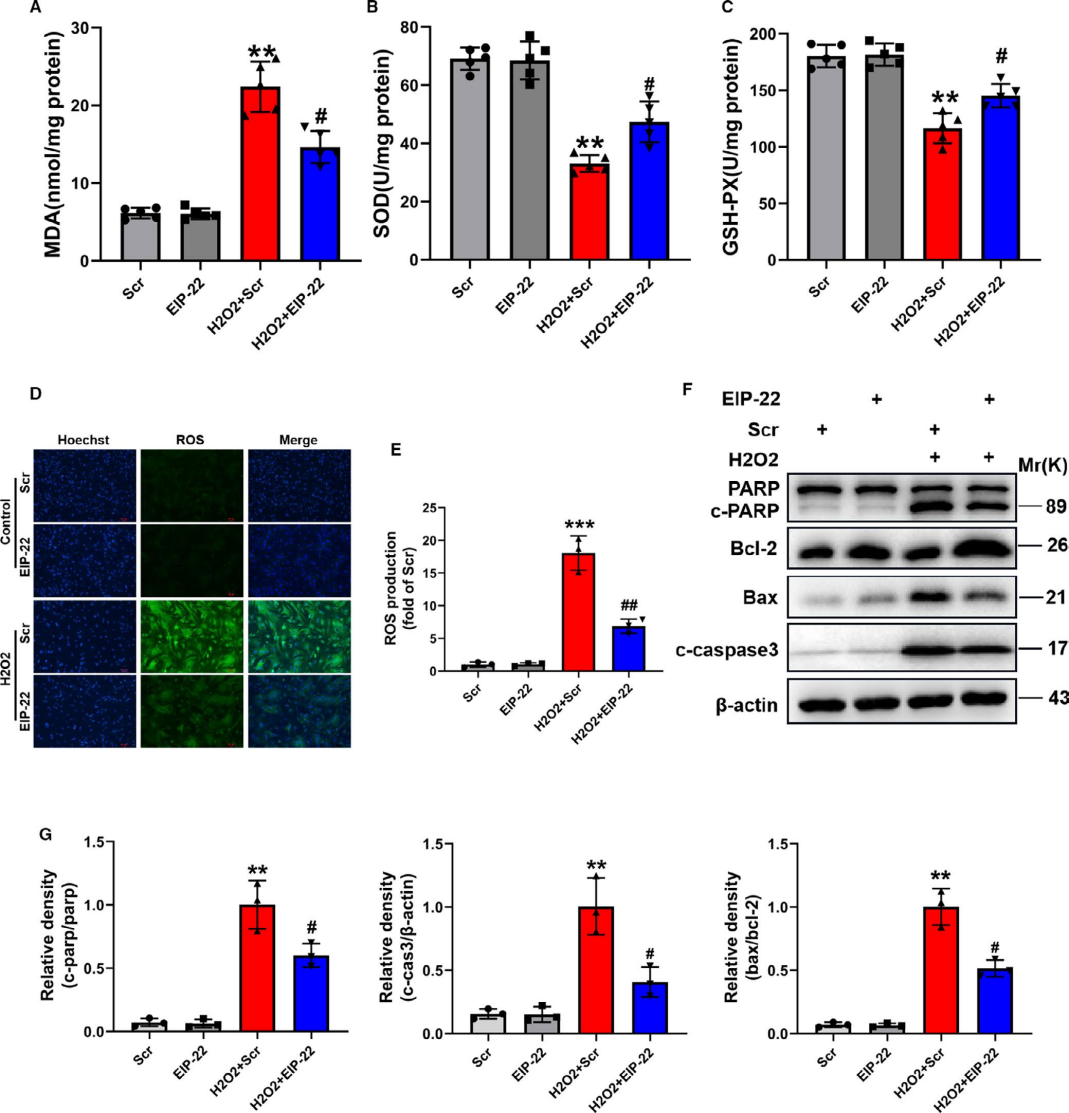
EIP‐22 attenuates oxidative stress and apoptosis in H_2_O_2_ mode. A, Cellular MDA content in H_2_O_2_ model. B, Cellular SOD activities in H_2_O_2_ model. C, Cellular GSH‐Px activities in H_2_O_2_ model. D, Representative images of ROS staining in H_2_O_2_ model. DCFH‐DA was used to detect intracellular ROS. Green, ROS. Blue, Hoechst. E, Quantification analysis of ROS. F, The proteins related to apoptosis were measured by Western blot in H_2_O_2_ model. G, Quantitative analysis of relative protein expression of PARP, cleaved‐caspase, Bax and Bcl‐2. The data represent means ± SD. ^**^ *P* < .01 vs. the Scr group, ^***^ *P* < .001 vs. the Scr group, ^#^ *P* < .05 vs. the H_2_O_2_ + Scr group, ^##^ *P* < .01 vs. the H_2_O_2_ + Scr group

To further improve the cardioprotective function of EIP‐22 in vitro, we demonstrated its effect on H_2_O_2_ induced myocardial injury. Similarly, oxidative stress was evaluated, reductions in the content of MDA (Figure 3A), increase in the activities of SOD and GSH‐Px (Figure 3B,C), and ROS was detected (Figure 3D,E). In addition, EIP‐22 also significantly decreased cleaved‐PARP activation, cleaved caspase‐3 and Bax protein level and increased Bcl‐2 protein level in H_2_O_2_ model (Figure 3F‐I).

### 3.4 EIP‐22 ameliorates myocardial I/R injury

**FIGURE 4:**
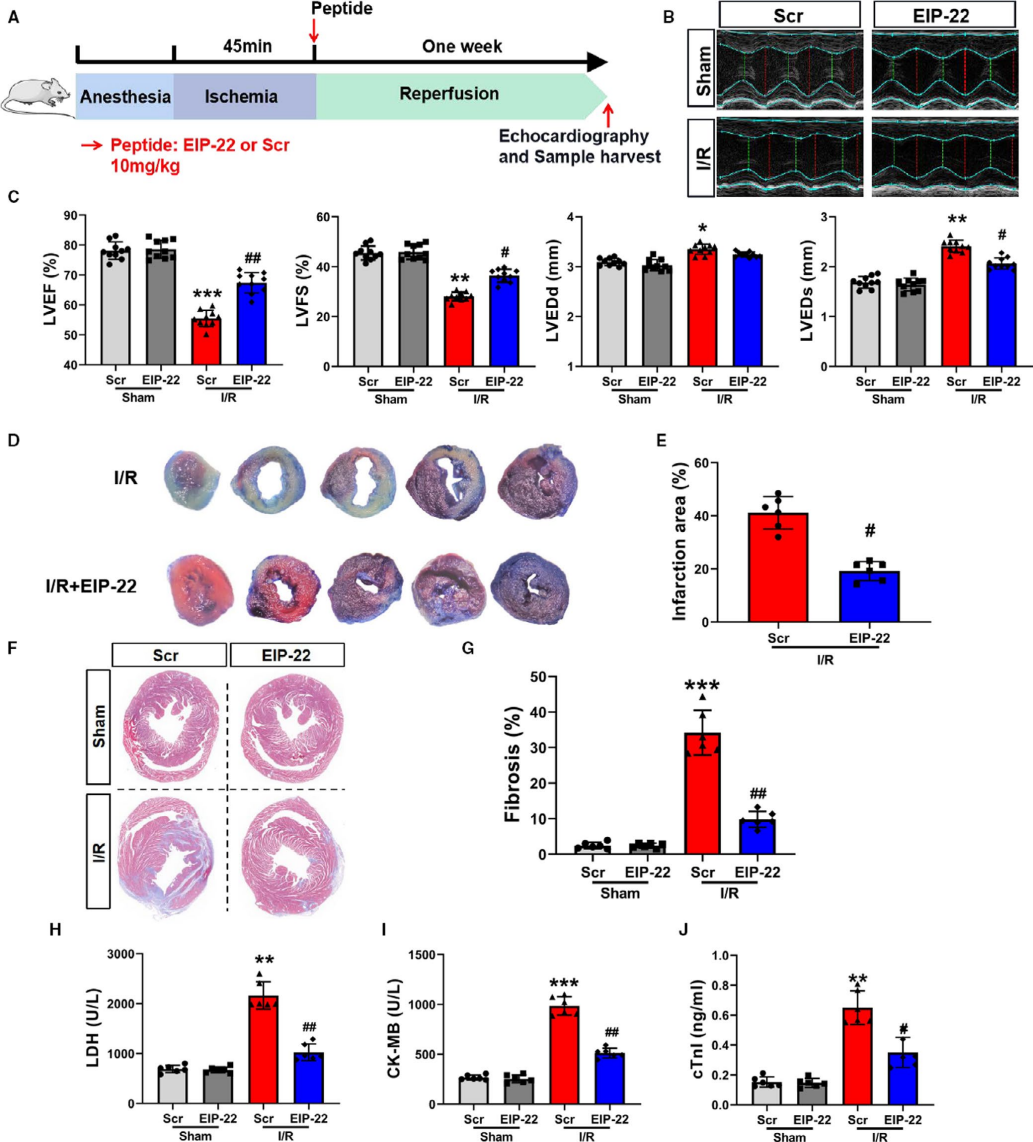
EIP‐22 ameliorates myocardial I/R injury. A, Myocardial I/R model diagram. LAD for 45 min, reperfusion for 1 wk, the concentration of EIP‐22 peptide is 10 mg/kg. B, The representative photographs of echocardiography. C, Quantification data of echocardiography analysis. D, The representative photographs of Evans Blue‐TTC staining. E, Quantification data of TTC staining. F, The representative photographs of Masson staining. G, Quantification data of Masson staining. H, The detection of serum LDH level. I, The detection of serum CK‐MB level. J, The detection of serum cTnI level. The data represent means ± SD. ^*^ *P* < .05 vs. the Sham + Scr group, ^**^ *P* < .01 vs. the Sham + Scr group, ^***^ *P* < .001 vs. the Sham + Scr group, ^#^ *P* < .05 vs. the I/R + Scr group, ^##^ *P* < .01 vs. the I/R + Scr group

To study the effect of EIP‐22 in vivo, we established an myocardial I/R injury model by ligating the left anterior descending (LAD) coronary artery for 45 minutes, followed by reperfusion for 1 week. The EIP‐22 peptide was given through tail vein before reperfusion (Figure 4A). The injection of EIP‐22 peptide protected the heart from I/R injury, as demonstrated by improved cardiac function assessed by echocardiography analysis (Figure 4B,C). The myocardial infarction area was evaluated through the Evans Blue‐TTC double staining. As shown in Figure 4D,E, myocardial infarction area was significantly decreased in the EIP‐22 peptide injection group. Moreover, Masson staining result showed that EIP‐22 peptide alleviated cardiac fibrosis (Figure 4F,G). Lastly, serum LDH, CK‐MB and cTnI levels were reduced significantly in the EIP‐22 peptide injection group (Figure 4H‐J). These data indicated that EIP‐22 had a cardioprotective effect on myocardial I/R injury in vivo.

### 3.5 EIP‐22 activates JAK2/STAT3 signalling pathway

**FIGURE 5:**
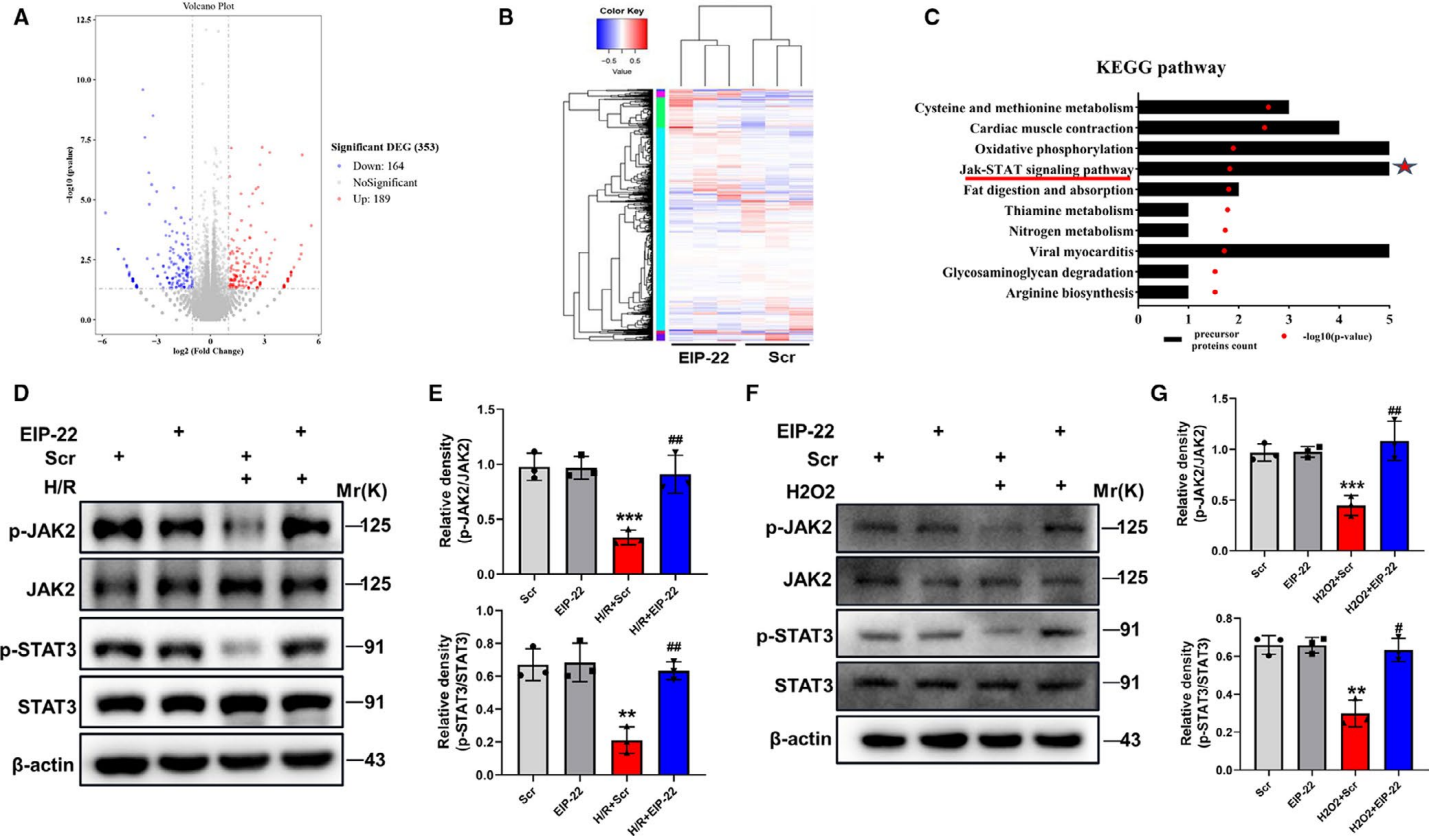
EIP‐22 activates JAK2/STAT3 signalling pathway. A, The RNA‐seq approach was used to map the transcriptome after EIP‐22 treatment in H/R model. The dysregulated genes were shown in the volcano plot, *P*‐value < .05 and fold change >2. B, Heat map of the differentially expressed genes. Red indicates up‐regulation, and green indicates down‐regulation. C, KEGG pathway analysis. D, The main proteins involved in JAK2/STAT3 signalling pathway were analysed by Western blot in H/R model. E, Quantification data of Western blot analysis. F, The main proteins involved in JAK2/STAT3 signalling pathway were analysed by Western blot in H_2_O_2_ model. G, Quantification data of Western blot analysis. The data represent means ± SD. ^**^ *P* < .01 vs. the Scr group, ^***^ *P* < .001 vs. the Scr group, ^#^ *P* < .05 vs. the H/R + Scr or H_2_O_2_ + Scr group, ^##^ *P* < .01 vs. the H/R + Scr or H_2_O_2_ + Scr group

To elucidate the potential mechanism of EIP‐22 in cardioprotection, the RNA‐seq approach was used to map the transcriptome after EIP‐22 treatment in H/R model.^23^ A total of 353 genes showed significant differences (*P* < .05, fold change > 2), of which 189 genes were up‐regulated and 164 genes were down‐regulated compared to H/R group (Figure 5A,B). KEGG pathway analysis was shown in Figure 5C, in which JAK / STAT signalling pathway attracted our attention because of their close relation to myocardial I/R injury. Growing evidence demonstrated that JAK/STAT signalling pathway, particularly JAK2/STAT3 signalling pathway, plays a key role in mediating protection of myocardium from I/R injury.^20^ A previous study revealed that post‐conditioning can attenuate myocardial apoptosis during long reperfusion via JAK2‐STAT3/Bcl‐2 signalling pathway.^24^ Therefore, we asked whether EIP‐22 peptide plays a protective role in the heart via regulating JAK2/STAT3 signalling pathway. Next, the main proteins involved in JAK2/STAT3 signalling pathway were analysed by Western blot, and the results showed that the H/R‐induced downward trend of phosphorylated JAK2 and phosphorylated STAT3 was significantly inhibited by EIP‐22 treatment (Figure 5D,E). Similar effects were observed in the H_2_O_2_ model (Figure 5F,G). The above results confirmed our conjecture that EIP‐22 peptide plays a protective role in the heart via activating JAK2/STAT3 signalling pathway.

### 3.6 AG490 eliminates the cardioprotection of EIP‐22

**FIGURE 6:**
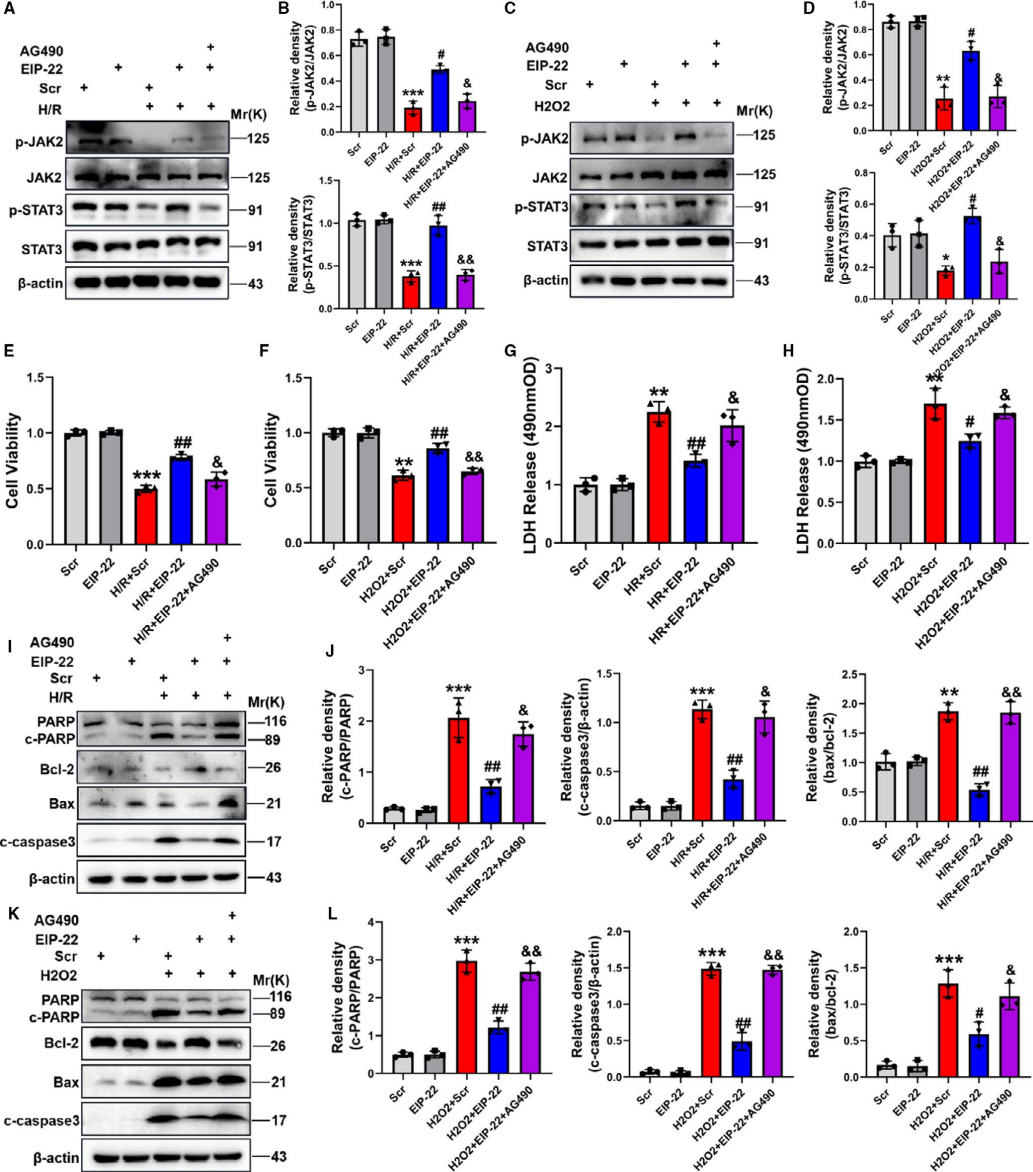
AG490 eliminates the cardioprotection of EIP‐22. A, The protein levels of p‐JAK2 and p‐STAT3 were inhibited whereas cells were treated with AG490 in H/R model. B, Quantification data of expression level of p‐JAK2 and p‐STAT3 in H/R model. C, The protein levels of p‐JAK2 and p‐STAT3 were inhibited whereas cells were treated with AG490 in H_2_O_2_ model. D, Quantification data of expression level of p‐JAK2 and p‐STAT3 in H_2_O_2_ model. E, The detection of cell viability in H/R model. F, The detection of cell viability in H_2_O_2_ model. G, The detection of LDH level in H/R model. H, The detection of LDH level in H_2_O_2_ model. I, The proteins related to apoptosis were detected in H/R model by Western blot analysis. J, Quantification data of Western blot analysis. K, The proteins related to apoptosis were detected in H_2_O_2_ model by Western blot analysis. L, Quantification data of Western blot analysis. The data represent means ± SD. ^**^ *P* < .01 vs. the Scr group, ^***^ *P* < .001 vs. the Scr group, ^#^ *P* < .05 vs. the H/R + Scr or H_2_O_2_ + Scr group, ^##^ *P* < .01 vs. the H/R + Scr or H_2_O_2_ + Scr group, ^&^ *P* < .05 vs. the H/R + EIP‐22 or H_2_O_2_ + EIP‐22 group, ^&&^ *P* < .01 vs. the H/R + EIP‐22 or H_2_O_2_ + EIP‐22 group

To further confirm that EIP‐22 peptide protected cardiomyocytes from H/R‐ and H_2_O_2_‐induced injury through activating JAK2/STAT3 signalling pathway, AG490, a selective inhibitor of JAK2/STAT3 signalling pathway, was used for rescue experiments.^25^ As shown in Figure 6, the protein levels of p‐JAK2 and p‐STAT3 were inhibited whereas cells were treated with AG490 (Figure 6A‐D). Then, we conducted a comprehensive rescue experiment to verify our hypothesis. The results showed that AG490 eliminated the protective effect of EIP‐22 on the cell viability and LDH release level in H/R and H_2_O_2_ models (Figure 6E‐H). Lastly, apoptosis was detected and the Western blot results showed that the activation of cleaved‐PARP, cleaved caspase‐3 and Bax were increased and Bcl‐2 was decreased in the AG490 and EIP‐22 cotreatment group (Figure 6I‐L). These results suggested that AG490 could rescue the cardioprotective effect of EIP‐22 and further demonstrated the hypothesis that EIP‐22 peptide exert cardioprotective effect via activating JAK2/STAT3 signalling pathway.

## 4 DISCUSSION

In the present study, we performed comprehensive analysis the function of a novel exercise‐induced peptide EIP‐22 in myocardial I/R injury. We found that EIP‐22 could protect cardiomyocytes from I/R injury, as demonstrated by increased cell viabilities and decreased apoptosis rates, ROS accumulation and LDH release in vitro. In vivo, we used mice I/R model to evaluate the function of EIP‐22. Our results demonstrated that EIP‐22 intervention could significantly reduce the cardiac damage, as indicated by decrease of myocardial infarction area, myocardial fibrosis and the serum level of CK‐MB and cTnI. Echocardiography analysis also showed that EIP‐22 could improve the cardiac function upon I/R injury. Mechanistic studies showed that EIP‐22 could increase the phosphorylation of JAK2 and STAT3. Further rescue experiments also revealed that EIP‐22 protected from myocardial I/R injury through JAK2/STAT3. This study provided evidence that a novel exercise‐induced peptide EIP‐22 might be a new candidate molecule for the treatment of myocardial injury induced by ischaemia‐reperfusion.

Exercise has been shown to play an important role in various physiological process. Studies have shown the beneficial effects of exercise on the obesity and cardiovascular disease.^26^, ^27^ However, the specific mechanism of exercise in such process was still unknown. Previous studies have shown that exercise‐induced exosomal miR‐342‐5p can protect heart against myocardial infarction.^28^ Ghrelin was a hormone which was induced by exercise and possessed an anti‐apoptosis effect.^20^ Similar, proteomics analysis also showed that protein was significantly changed during exercise.^29^ Thus, we wondered to explore more components during exercise, which provide a better understanding for the beneficial effects of exercise.

It was well known that oxidative stress and apoptosis were the main events of myocardial injury.^30^ Increased oxidative stress in the body can aggravate myocardial injury, and increased intracellular ROS accumulation can cause mitochondrial dysfunction, further aggravate myocardial apoptosis, and finally affect the process of myocardial remodelling.^31^ Studies have shown that H_2_O_2_ can increase the production of ROS, induce oxidative stress injury and myocardial apoptosis in cardiomyocytes, which is a classic model to simulate the oxidative damage induced by hypoxia in vitro.^32^ H/R is an important in vitro model for simulating myocardial ischaemia‐reperfusion injury. In our study, we successfully identified a exercise‐induced peptide named EIP‐22. To assess the function of EIP‐22, we established H/R model and H_2_O_2_ model to simulate ischaemia‐reperfusion injury and oxidative stress injury in vitro. Our study showed that EIP‐22 protected cardiomyocytes by reducing production of ROS, apoptosis rates and activation of apoptosis related proteins PARP, cleaved caspase‐3 and Bax. Similarly, the long non‐coding RNA Hotair reduced oxidative stress and cardiomyocytes apoptosis against ischaemia‐reperfusion injury.^33^ In addition, cyclosporine A attenuated oxidative stress induced cardiomyocytes apoptosis through alleviating the production of ROS and up‐regulating heat shock protein 70.^34^ Therefore, from the perspective of anti‐oxidative stress and anti‐apoptosis, we may find the effective therapeutic target of myocardial injury caused by ischaemia‐reperfusion.

The JAK/STAT signalling pathway is mainly composed of the JAKs protein family and the STATs protein family, among which JAK is the upstream kinase of STAT. The JAK/STAT signalling pathway is widely involved in a variety of biological effects such as cell stress, differentiation, proliferation and apoptosis.^35^, ^36^ JAK2/STAT3 is one of the important members of JAK/STAT signalling pathway. Accumulating studies have confirmed that JAK2/STAT3 signalling pathway plays a core regulatory role in myocardial I/R injury and the activation of JAK2/STAT3 signalling pathway can significantly up‐regulate the synthesis of mitochondrial antioxidant enzymes, reduce the production of hydrogen peroxide and alleviate the mitochondria oxidative stress injury and cardiomyocyte apoptosis.^37^, ^38^ However, the role of JAK2/STAT3 signalling pathway in the myocardial protection mediated by EIP‐22 has not been reported. In this study, for the first time, we confirmed that EIP‐22 peptide could up‐regulate the expression of phosphorylated JAK2 and STAT3 in H/R and H_2_O_2_ models and activate JAK2/STAT3 signalling pathway to exert myocardial protective effect. In addition, as an inhibitor of the JAK2/STAT3 signalling pathway, AG490 eliminated the myocardial protective effects of EIP‐22. Our results further supported the important role of JAK/STAT signalling pathway in the myocardial protective effects of EIP‐22 on ischaemia‐reperfusion injury.

Although we identified a novel exercise‐induced peptide, which we named as EIP‐22, protect cardiomyocytes from I/R injury and oxidative stress, there were still some limitations in our study. For example, whether different modification methods could influence the function of EIP‐22 remains to be verified. In addition, the effect of EIP‐22 peptide on other kinds of cardiomyocytes needs to be clarified in the future, such as H9c2 cells and AC16 cells. Therefore, in future work, we will evaluate the effect of EIP‐22 on other types of cardiomyocytes and make different modifications to clarify its cardioprotective function.

## 5 CONCLUSION

In summary, this is the first study that we verified the function and mechanism of EIP‐22 in myocardial I/R injury. Our study provided a new insight into treatment strategy of myocardial I/R injury.

## CONFLICT OF INTERESTS

The authors declare no conflicts of interest.

## AUTHOR CONTRIBUTION

Li Zhang: Data curation (equal); Formal analysis (equal); Project administration (equal); Resources (equal); Writing‐original draft (equal). Xuejun Wang: Data curation (equal); Project administration (equal). Hao Zhang: Data curation (equal); Project administration (equal); Validation (equal). Mengwen Feng: Project administration (equal). Jingjing Ding: Project administration (equal). Bing Zhang: Project administration (equal). Zijie Cheng: Conceptualization (equal); Supervision (equal); Visualization (equal). lingmei qian: Conceptualization (equal); Data curation (equal); Funding acquisition (equal); Supervision (equal).

## ETHICAL APPROVAL

All mice experiments were conducted according to the Guide for the Care and Use of Laboratory Animals published by the National Institutes of Health (NIH Publications No. 85‐23, revised 1996) and reviewed by the Animal Experiment Ethics Committee of Nanjing Medical University (Nanjing, China).

## Data Availability

The data that support the findings of this study are available from the corresponding author upon reasonable request.
